# Effect of the 2010 task force criteria on reclassification of cardiac MRI criteria for ARVC

**DOI:** 10.1186/1532-429X-16-S1-P270

**Published:** 2014-01-16

**Authors:** Ting Liu, Amit Pursnani, Umesh Sharma, Yongkasem Vorasettakarnkij, Daniel Verdini, Peerawut Deeprasertkul, Ashley M Lee, Heidi Lumish, Manavjot Sidhu, Hector Medina, Stephan Danik Danik, Godtfred Holmvang, Suhny Abbara, Udo Hoffmann, Brian B Ghoshhajra

**Affiliations:** 1 Cardiac MR PET CT Program, MGH, Boston, Massachusetts, USA; 2 Radiology, First hospital of China Medical University, shenyang, liaoning, China

## Background

We sought to evaluate the effect of application of the revised 2010 Task Force Criteria (TFC) on the prevalence of major and minor MRI (Magnetic Resonance Imaging) criteria for Arrythmogenic Right Ventricular Cardiomyopathy (ARVC) versus application of the original 1994 TFC. We also assessed the utility of MRI to identify alternative diagnoses for patients referred for ARVC evaluation.

## Methods

Nine-hundred-sixty-eight consecutive patients, referred to our institution for cardiac magnetic resonance imaging (CMR) with clinical suspicion of ARVC from 1995 to 2010, were evaluated for the presence of major and minor CMR criteria per the 1994 and 2010 ARVC TFC. MRI criteria included right ventricle (RV) dilatation, reduced RV ejection fraction, RV aneurysm, or regional RV wall motion abnormalities. Quantitative and qualitative RV measures of end diastolic volume (RVEDV) and RV ejection fraction (RVEF) were available in 45% and 85% of cases, respectively.

## Results

Of 968 patients, 220 (22.7%) fulfilled either a major or a minor 1994 TFC, and 25 (2.6%) fulfilled any of the 2010 TFC criterion. Among patients meeting any 1994 criteria, only 25 (11.4%) met at least one 2010 criterion. All patients who fulfilled a 2010 criteria also satisfied at least one 1994 criterion. Per the 2010 TFC, 21 (2.2%) patients met a major criteria and 4 (0.4%) patients fulfilled at least one minor criterion. Eight patients meeting 1994 minor criteria were reclassified as satisfying 2010 major criteria, while 4 patients fulfilling 1994 major criteria were reclassified to only minor or no criteria under the 2010 TFC. Eighty-nine (9.2%) patients had alternative cardiac diagnoses, including 43 (4.4%) with clinically significant potential ARVC mimics. These included cardiac sarcoidosis, RV volume overload conditions (e.g. ASD, PAPVR), and other cardiomyopathies.

## Conclusions

Application of the 2010 TFC resulted in reduction of total patients meeting any diagnostic MRI criteria for ARVC from 22.7% to 2.5% versus the 1994 TFC. The inclusion of myocardial fat would not have changed the diagnosis in any patients. CMR identified alternative cardiac diagnoses in 9.2% of patients, and 4.4% of the diagnoses were potential mimics of ARVC.

## Funding

None.

**Table 1:** Comparison of 1994 and 2010 MRI criteria for ARVC

2010	1994		
	
	Major or Minor	None	total
Major or Minor	25	0	25

None	195	748	943

total	220	748	968

**Figure 1:**
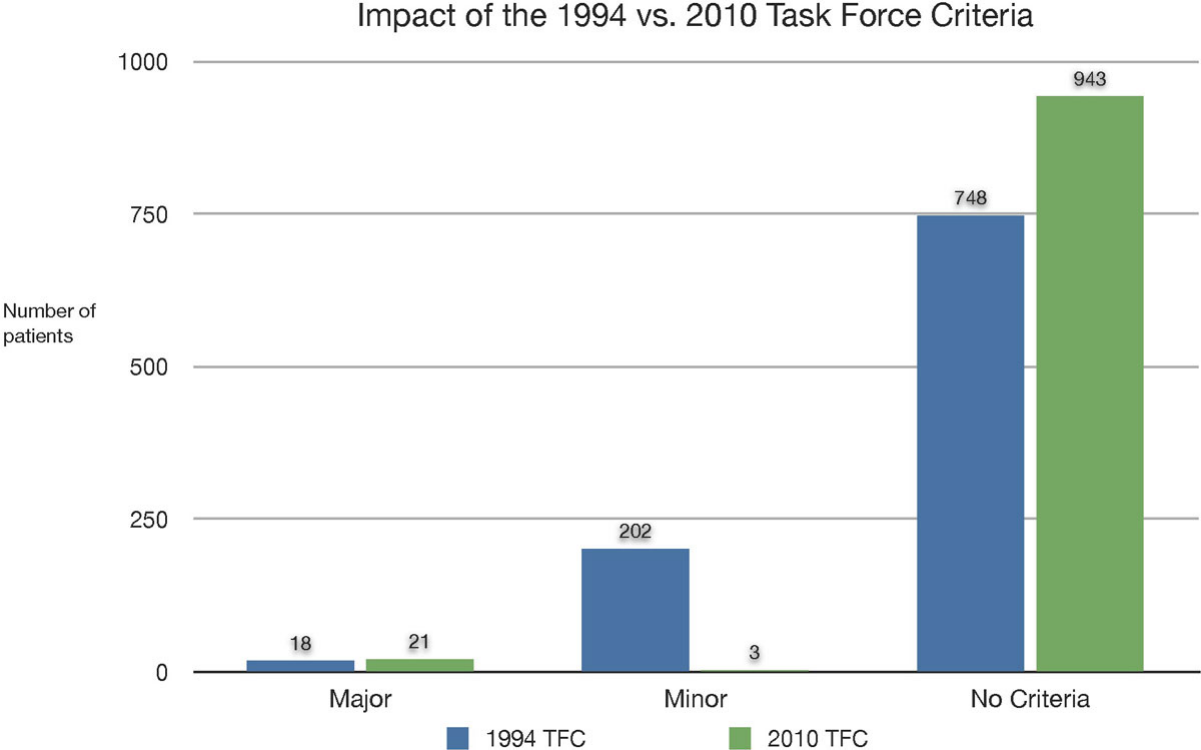
**Incidence of 1994 and 2010 ARVD Criteria The graph shows the different incidence of patients with major criteria, minor criteria, or no criteria according to 1994 cardiac magnetic resonance criteria (blue bars) and 2010 criteria (green bars)**.

